# Supplementary material for: Coral larval aquaculture: Species-specific survival and microbial dynamics in flow-through systems
Source: PLoS One. 2026 Feb 13;21(2):e0340422. doi: 10.1371/journal.pone.0340422 (PMC12904410; doi:10.1371/journal.pone.0340422)
Supplement: S1 Fig — Points represent means of culture tanks from each treatment. Triangular points distinguish tanks with surface agitation while dashed lines distinguish tanks at higher turnover. Gray or black points represent tanks without UV sterilization while pink or purple points represent tanks with UV sterilization, with darker shades (black and purple) representing tanks with at higher larval density. Blue dashed lines represent values in the incoming seawater before (dark blue) and after UV sterilization (light blue). The black vertical line distinguishes samples taken before and after larvae were added. (DOCX) [file pone.0340422.s001.docx]

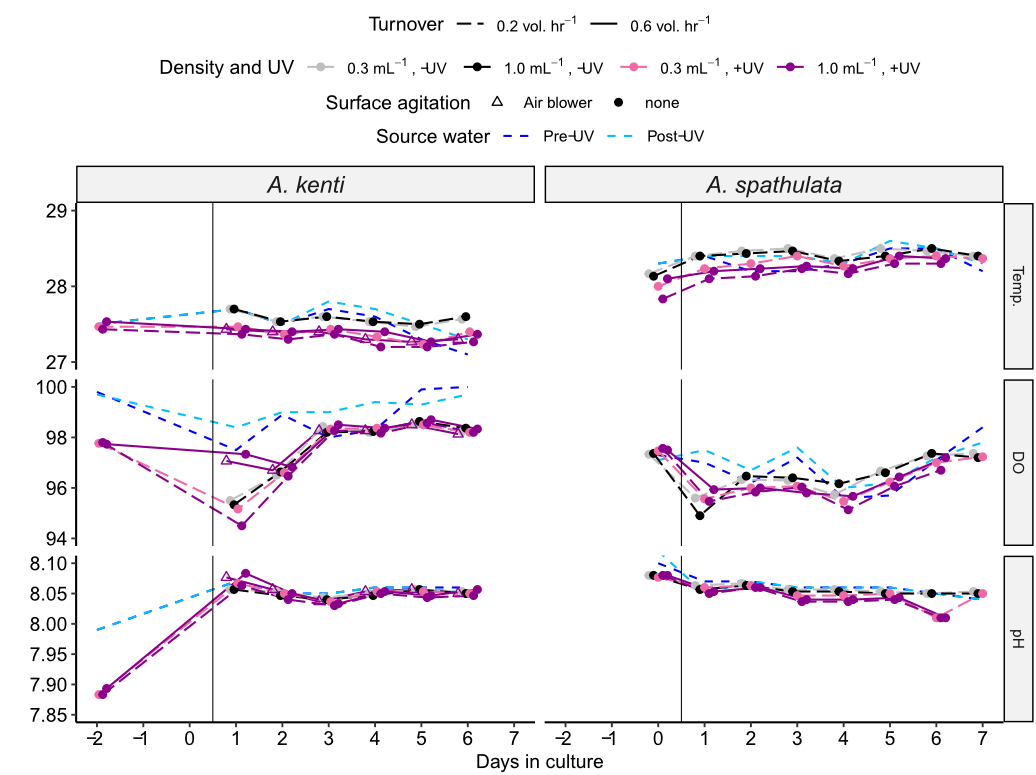


**S4 Fig. Daily temperature (°C), dissolved oxygen (DO; % saturation), and pH for cultures of *Acropora kenti* and *Acropora spathulata* larvae.** Points represent means of culture tanks from each treatment. Triangular points distinguish tanks with surface agitation while dashed lines distinguish tanks at higher turnover. Gray or black points represent tanks without UV sterilization while pink or purple points represent tanks with UV sterilization, with darker shades (black and purple) representing tanks with at higher larval density. Blue dashed lines represent values in the incoming seawater before (dark blue) and after UV sterilization (light blue). The black vertical line distinguishes samples taken before and after larvae were added.
