# Supplementary material for: Coral larval aquaculture: Species-specific survival and microbial dynamics in flow-through systems
Source: PLoS One. 2026 Feb 13;21(2):e0340422. doi: 10.1371/journal.pone.0340422 (PMC12904410; doi:10.1371/journal.pone.0340422)
Supplement: S2 Fig — Larvae are approximately 0.06 mm2. (DOCX) [file pone.0340422.s002.docx]

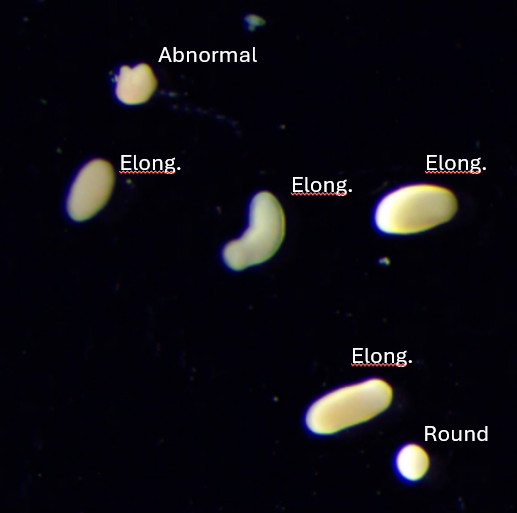


S2 Fig. Example *A. spathulata* larvae that are abnormal or normal (elongated, or round). Larvae are approximately 0.06 mm^2^.
