# Supplementary material for: Coral larval aquaculture: Species-specific survival and microbial dynamics in flow-through systems
Source: PLoS One. 2026 Feb 13;21(2):e0340422. doi: 10.1371/journal.pone.0340422 (PMC12904410; doi:10.1371/journal.pone.0340422)
Supplement: S3 Fig — The x-axis is water volume turnover (vol. hr-1) and the y-axis is the proportion of larvae settled. Colors distinguish the stocking density (light vs dark) and UV sterilization (shades of purple versus black) culture treatments. For A. kenti, surface agitation is shown using a triangle. (DOCX) [file pone.0340422.s003.docx]

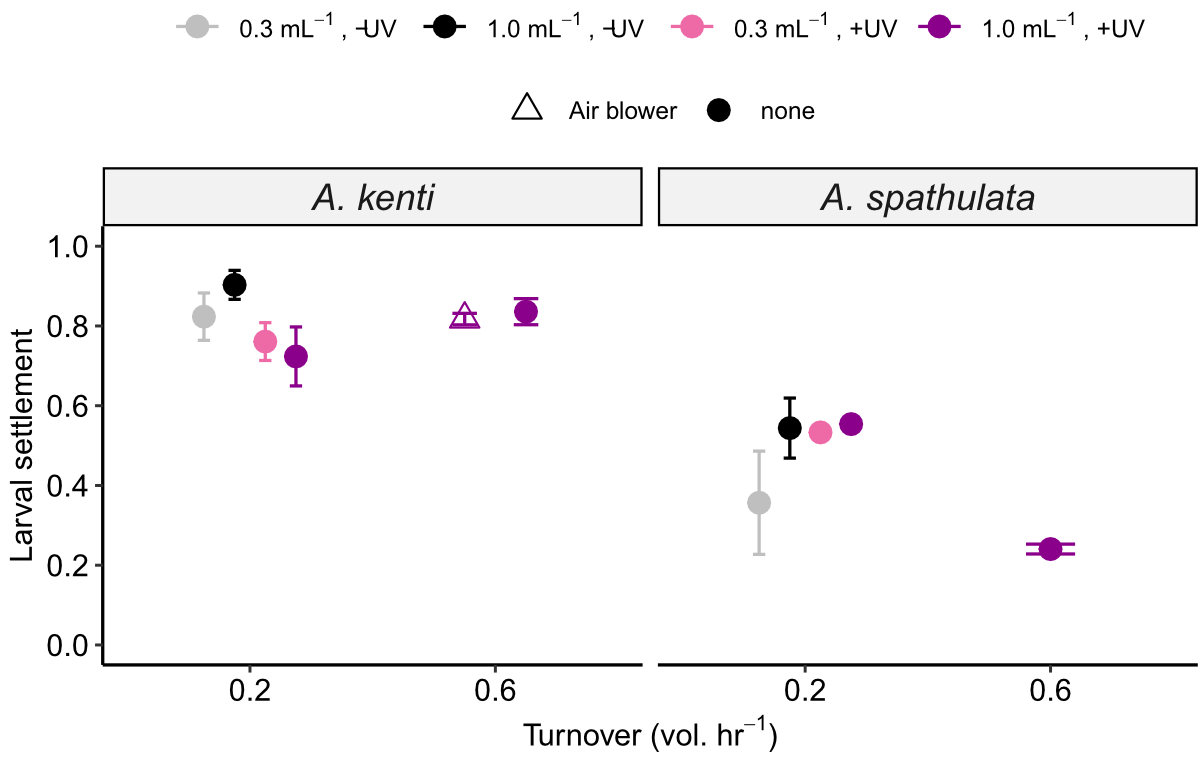


**S3 Fig. Larval settlement proportion for *A. kenti* and *A. spathulata* 5 days post-fertilization.** The x-axis is water volume turnover (vol. hr^-1^) and the y-axis is the proportion of larvae settled. Colors distinguish the stocking density (light vs dark) and UV sterilization (shades of purple versus black) culture treatments. For *A. kenti*, surface agitation is shown using a triangle.
