# Supplementary material for: Coral larval aquaculture: Species-specific survival and microbial dynamics in flow-through systems
Source: PLoS One. 2026 Feb 13;21(2):e0340422. doi: 10.1371/journal.pone.0340422 (PMC12904410; doi:10.1371/journal.pone.0340422)
Supplement: S4 Fig — Columns distinguish coral species. Larval stocking densities are represented using different colors, tank turnover treatments have solid or dashed lines, and the surface agitation treatment is denoted with triangles. Points represent means and error bars represent SE. Blue lines represent values in the incoming seawater before (dark blue) and after UV sterilization (light blue). The black vertical line distinguishes samples taken before and after larvae were added. (DOCX) [file pone.0340422.s004.docx]

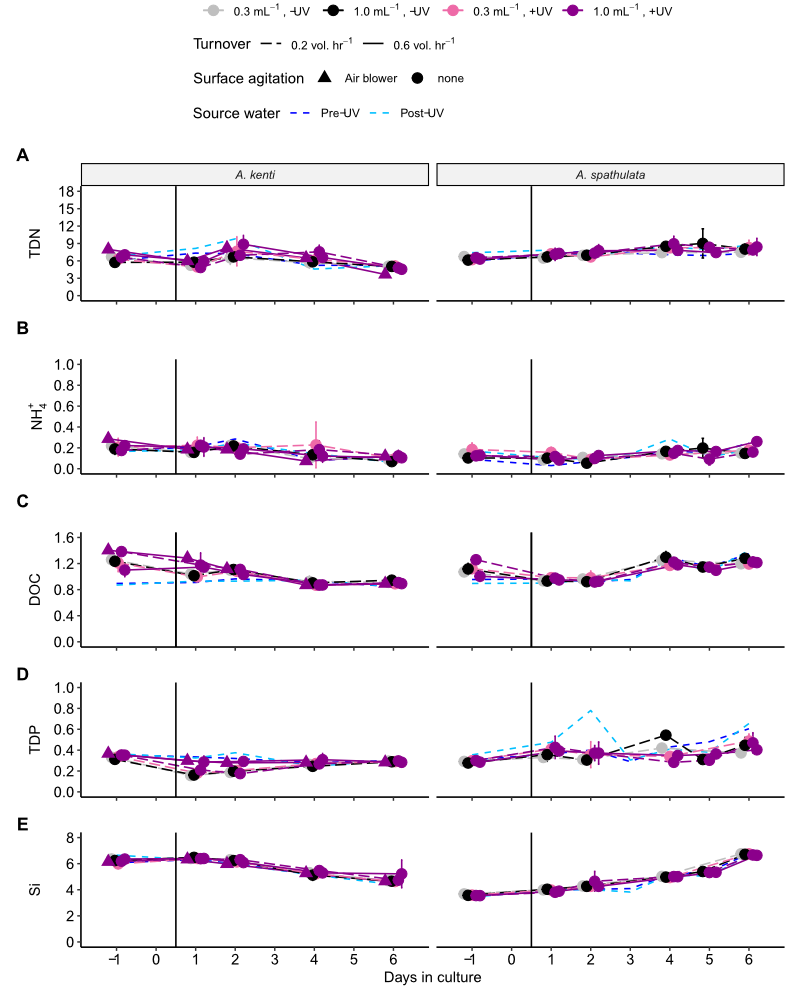


**S4 Fig. Nutrient levels in culture tanks including total dissolved N (TDN; µM), ammonium (**$\mathbf{NH}_{\boldsymbol{4}}^{\boldsymbol{+}}$**), dissolved organic carbon (DOC, mg L^-1^), total dissolved phosphorus (TDP; µmol L^-1^), and silica (Si; µmol L^-1^).** Columns distinguish coral species. Larval stocking densities are represented using different colors, tank turnover treatments have solid or dashed lines, and the surface agitation treatment is denoted with triangles. Points represent means and error bars represent SE. Blue lines represent values in the incoming seawater before (dark blue) and after UV sterilization (light blue). The black vertical line distinguishes samples taken before and after larvae were added.
