# Supplementary material for: Coral larval aquaculture: Species-specific survival and microbial dynamics in flow-through systems
Source: PLoS One. 2026 Feb 13;21(2):e0340422. doi: 10.1371/journal.pone.0340422 (PMC12904410; doi:10.1371/journal.pone.0340422)
Supplement: S5 Fig — Points and error bars represent mean and SE, respectively. The gray band in D indicates the mean±SE of bacterial abundance from local reef samples. Columns distinguish coral species. Larval stocking densities and UV sterilization are represented using different colors and tank turnover treatments have solid or dashed lines. The blue line represents values in the incoming after UV sterilization. The black vertical line indicates when larvae were added. (DOCX) [file pone.0340422.s005.docx]

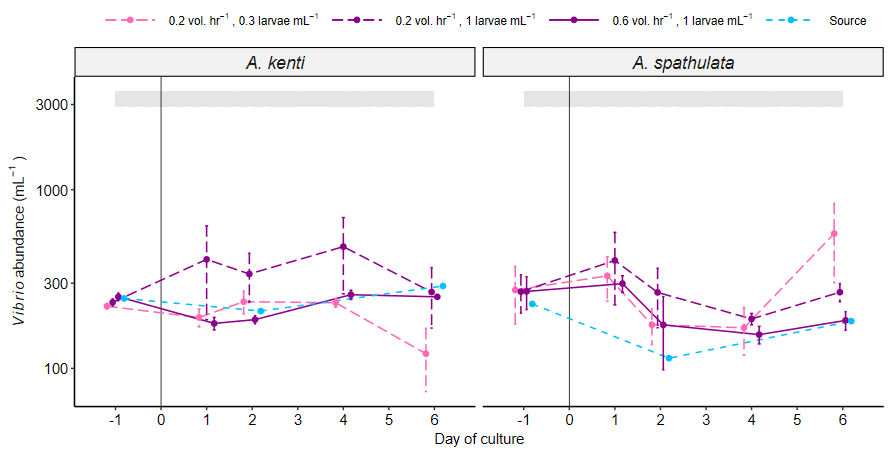


**S5 Fig. *Vibrio* spp. abundance (cells mL^-1^) measured using ddPCR. Points and error bars represent mean and SE, respectively.** The gray band in D indicates the mean±SE of bacterial abundance from local reef samples. Columns distinguish coral species. Larval stocking densities and UV sterilization are represented using different colors and tank turnover treatments have solid or dashed lines. The blue line represents values in the incoming after UV sterilization. The black vertical line indicates when larvae were added.
