# Supplementary material for: Coral larval aquaculture: Species-specific survival and microbial dynamics in flow-through systems
Source: PLoS One. 2026 Feb 13;21(2):e0340422. doi: 10.1371/journal.pone.0340422 (PMC12904410; doi:10.1371/journal.pone.0340422)
Supplement: S6 Fig — ASV abundances are grouped by the turnover treatments and genera. Points represent mean abundance for each ASV and error bars represent SE. (DOCX) [file pone.0340422.s006.docx]

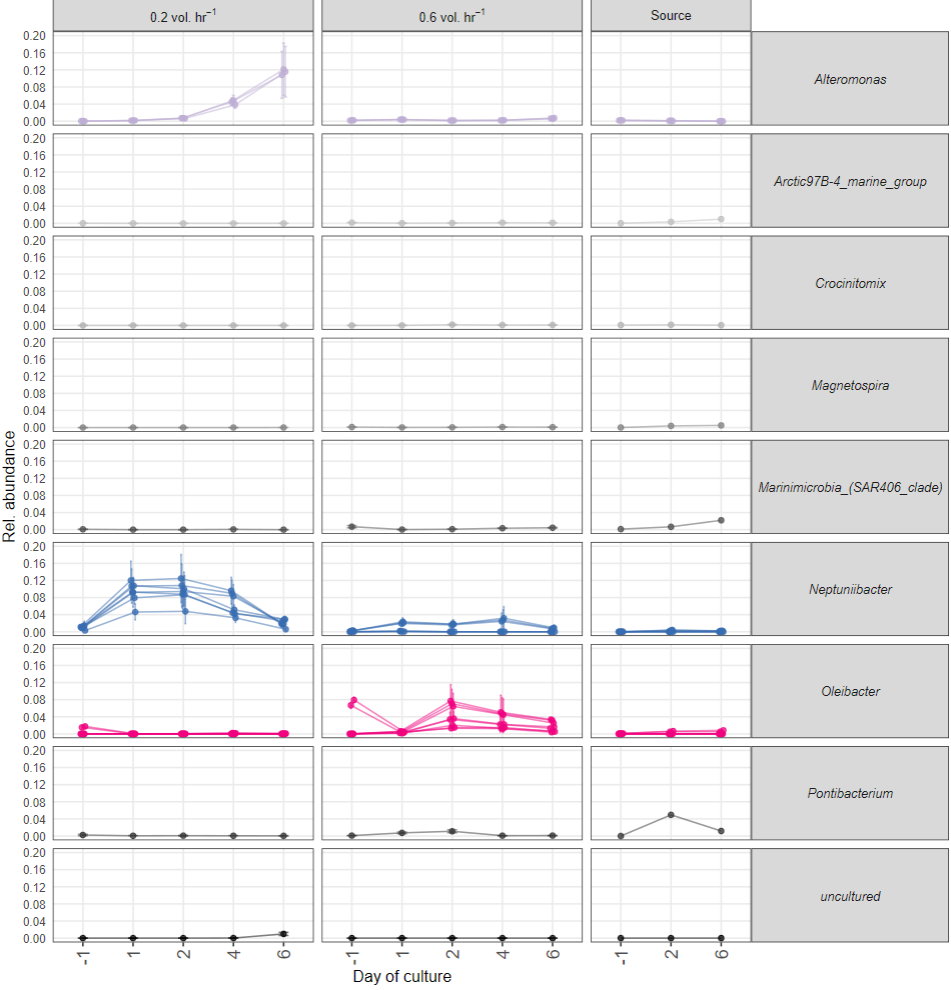


S6 Fig. Relative abundance of 24 ASV in *Acropora kenti* cultures with significantly different abundance between 0.2 and 0.6 vol. hr^-1^ treatments. ASV abundances are grouped by the turnover treatments and genera. Points represent mean abundance for each ASV and error bars represent SE.
