# Supplementary material for: Coral larval aquaculture: Species-specific survival and microbial dynamics in flow-through systems
Source: PLoS One. 2026 Feb 13;21(2):e0340422. doi: 10.1371/journal.pone.0340422 (PMC12904410; doi:10.1371/journal.pone.0340422)
Supplement: S7 Fig — ASV abundances are grouped by Phylum:Genus (ASV are unclassified where genus is not provided). The blue points and lines represent the abundance of the same ASV in the source water. Points represent mean abundance for each ASV and error bars represent SE. (DOCX) [file pone.0340422.s007.docx]

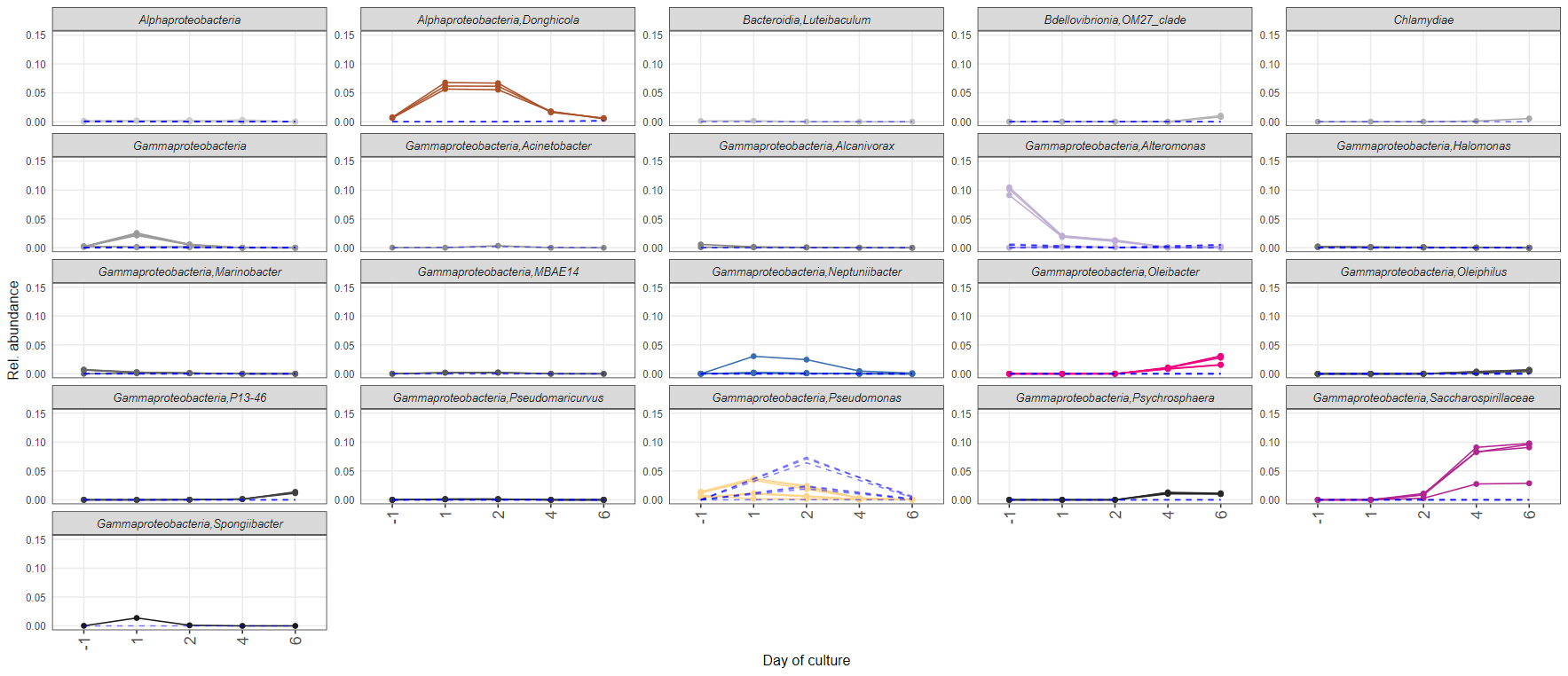


Supplemental Figure 7. Relative abundance of 76 ASV in *Acropora spathulata* cultures with significantly different abundance between Day 1 and Day 6 of culture. ASV abundances are grouped by Phylum:Genus (ASV are unclassified where genus is not provided). The blue points and lines represent the abundance of the same ASV in the source water. Points represent mean abundance for each ASV and error bars represent SE.
