# Supplementary material for: Coral larval aquaculture: Species-specific survival and microbial dynamics in flow-through systems
Source: PLoS One. 2026 Feb 13;21(2):e0340422. doi: 10.1371/journal.pone.0340422 (PMC12904410; doi:10.1371/journal.pone.0340422)
Supplement: S1 Table — (DOCX) [file pone.0340422.s008.docx]

S1 Table. Sampling schedule for different responses.

| Species | Response | Days of culture | | | | | | | | | |
| --- | --- | --- | --- | --- | --- | --- | --- | --- | --- | --- | --- |
|  |  | -2 | -1 | 0 | 1 | 2 | 3 | 4 | 5 | 6 | 7 |
| *A. kenti* | Larval characteristics |  |  |  | x | x | x | x | x | x | x |
|  | Temp., DO, pH | x |  |  | x | x | x | x | x | x | x |
|  | Nutrients |  | x |  | x | x |  | x |  | x |  |
|  | Microbial community |  | x |  | x | x |  | x |  | x |  |
| *A. spathulata* | Larval characteristics |  |  |  | x | x | x | x | x | x | x |
|  | Temp., DO, pH |  |  | x | x | x | x | x | x | x | x |
|  | Nutrients |  | x |  | x | x |  | x |  | x |  |
|  | Microbial community |  | x |  | x | x |  | x | x | x |  |

d
