# Supplementary material for: Coral larval aquaculture: Species-specific survival and microbial dynamics in flow-through systems
Source: PLoS One. 2026 Feb 13;21(2):e0340422. doi: 10.1371/journal.pone.0340422 (PMC12904410; doi:10.1371/journal.pone.0340422)
Supplement: S2 Table — For responses with significant treatment*day interactions, all treatments were compared using Tukey post hoc comparisons. From these comparisons, we interpret the results of 6 pairwise comparisons for Acropora kenti and 5 for Acropora spathulata that differed in a single culture treatment condition (Tested treatment). Two comparisons are used to test differences due to stocking density and sterilization and are denoted using superscript ab and cd, respectively. The controlled treatments represent the shared culture conditions for each comparison. (DOCX) [file pone.0340422.s009.docx]

S2 Table. Post hoc comparisons. For responses with significant treatment*day interactions, all treatments were compared using Tukey post hoc comparisons. From these comparisons, we interpret the results of 6 pairwise comparisons for *Acropora kenti* and 5 for *Acropora spathulata* that differed in a single culture treatment condition (Tested treatment). Two comparisons are used to test differences due to stocking density and sterilization and are denoted using superscript ^ab^ and ^cd^, respectively. The controlled treatments represent the shared culture conditions for each comparison.

| Species | Tested treatment | Comparison |  | Controlled treatments | | |
| --- | --- | --- | --- | --- | --- | --- |
|  |  |  |  | Density (mL^-1^) | Turnover  (vol. hr^-1^) | UV |
| *A. kenti* | Stocking density | 0.3 vs 1.0 larvae ml^-1^ | ^a^ |  | 0.2 | -UV |
|  | Stocking density | 0.3 vs 1.0 larvae ml^-1^ | ^b^ |  | 0.2 | +UV |
|  | Turnover | 0.2 vs 0.6 vol. hr^-1^ |  | 1.0 |  | +UV |
|  | Sterilization | +/- UV | ^c^ | 0.3 | 0.2 |  |
|  | Sterilization | +/- UV | ^d^ | 1.0 | 0.2 |  |
|  | Surface agitation | +/- air blower |  | 1.0 | 0.6 | +UV |
| *A. spathulata* | Stocking density | 0.3 vs 1.0 larvae ml^-1^ | ^a^ |  | 0.2 | -UV |
|  | Stocking density | 0.3 vs 1.0 larvae ml^-1^ | ^b^ |  | 0.2 | +UV |
|  | Turnover | 0.2 vs 0.6 vol. hr^-1^ |  | 1.0 |  | +UV |
|  | Sterilization | +/- UV | ^c^ | 0.3 | 0.2 |  |
|  | Sterilization | +/- UV | ^d^ | 1.0 | 0.2 |  |
