# Supplementary material for: Coral larval aquaculture: Species-specific survival and microbial dynamics in flow-through systems
Source: PLoS One. 2026 Feb 13;21(2):e0340422. doi: 10.1371/journal.pone.0340422 (PMC12904410; doi:10.1371/journal.pone.0340422)
Supplement: S3 Table — The table includes the response variables and the significant differences between consecutive days (e.g., Day 1 – Day −1, Day 2 – Day 1, etc). Each cell contains an effect size (difference) and an associated statistical significance (p value). Empty cells indicate pairwise comparisons with p > 0.05 while dashes indicate days where a given response was not measured. Responses without significant differences on any day are not shown. * this difference in Si was measured on Day 6 – Day 4. (DOCX) [file pone.0340422.s010.docx]

S3 Table. Significant post hoc comparisons for main effects of time (Table 2, Table 3) for larval appearance, water temperature, silica (Si) concentrations, and Faith’s phylogenetic diversity. The table includes the response variables and the significant differences between consecutive days (e.g., Day 1 – Day -1, Day 2 – Day 1, etc). Each cell contains an effect size (difference) and an associated statistical significance (*p* value). Empty cells indicate pairwise comparisons with *p*>0.05 while dashes indicate days where a given response was not measured. Responses without significant differences on any day are not shown. * this difference in Si was measured on Day 6 - Day 4

| Species | Response | Days 1 – (-1) | Days 2–1 | Days 3-2 | Days 4-3 | Days 5-4 | | Days 6-5 |
| --- | --- | --- | --- | --- | --- | --- | --- | --- |
| *A. kenti* | Appearance | - |  |  | 1.9× (*p*≤0.01) |  |  | |
|  | Temperature | - | -0.09 (*p*≤0.01) | 0.05 (*p*≤0.01) | -0.09 (*p*≤0.01) | -0.06 (*p*≤0.01) | | 0.09 (*p*≤0.01) |
|  | Silica | 0.19 (*p*≤0.01) | -0.22 (*p*=0.01) | - | -0.90 (*p*≤0.01) | - | | -0.62 (*p*≤0.01)* |
|  | Faith phyl. div. | -7.53 (*p*≤0.01) | 5.84 (*p*=0.04) | - | 8.96 (*p*≤0.01) | - | |  |
| *A. spathulata* | Survival | - |  |  | -0.13 (*p*≤0.01) |  | | -0.14 (*p*≤0.01) |
|  | Faith phyl. div. |  | 5.49 (*p*≤0.01) | - | -14.55 (*p*≤0.01) | - | |  |
