# Supplementary material for: Coral larval aquaculture: Species-specific survival and microbial dynamics in flow-through systems
Source: PLoS One. 2026 Feb 13;21(2):e0340422. doi: 10.1371/journal.pone.0340422 (PMC12904410; doi:10.1371/journal.pone.0340422)
Supplement: S4 Table — The table reports the species, the measured response, treatments being compared, and the significant effects on each experimental day. Each cell contains an effect size (difference or ratio) and statistical significance (p value). Empty cells indicate pairwise comparisons with p > 0.05 while dashes indicate days where a given response was not measured. Responses without significant differences on any day are not shown. b refers to the comparison of stocking densities in tanks with UV sterilization in S2 Table. (DOCX) [file pone.0340422.s011.docx]

S4 Table. Significant post hoc comparisons for treatment*time interactions on larval, bacterial, and *Vibrio* responses (Table 2). The table reports the species, the measured response, treatments being compared, and the significant effects on each experimental day. Each cell contains an effect size (difference or ratio) and statistical significance (p value). Empty cells indicate pairwise comparisons with p>0.05 while dashes indicate days where a given response was not measured. Responses without significant differences on any day are not shown. ^b^ refers to the comparison of stocking densities in tanks with UV sterilization in Supplementary Table 2.

| Species | Response | Comparison | Day -1 | Day 1 | Day 2 | Day 3 | Day **4** | Day **5** | Day **6** | Day **7** |
| --- | --- | --- | --- | --- | --- | --- | --- | --- | --- | --- |
| *A. kenti* | Size (mm^2^) | All non-sig. | - |  |  |  |  |  |  |  |
|  | Bact. abund. (cells mL^-1^) | Stock. density (1.0/0.3 ml^-1^) | 2.4× (*p*≤0.01)^b^ |  |  | - |  | - |  | - |
|  | Bact. abund. (cells mL^-1^) | Turnover (0.6/0.2 vol. hr^-1^) | 0.4× (*p*≤0.01) | 0.3× (*p*≤0.01) | 0.4× (*p*≤0.01) | - | 0.5× (*p*≤0.01) | - |  | - |
|  | *Vibrio* abund. (cells mL^-1^) | Stock. density (1.0/0.3 mL^-1^) |  |  |  | - |  | - | 1.5 (*p*=0.01)^b^ | - |
|  | *Vibrio* abund. (cells mL^-1^) | Turnover (0.6/0.2 vol. hr^-1^) |  |  | 0.8× (*p*=0.03) | - |  | - | 1.4× (*p*=0.03) | - |
| *A. spa* | Size (mm^2^) | Stock. Density (1.0 – 0.3 mL^-1^) |  |  |  |  | -0.02 (*p*≤0.01)^b^ |  |  |  |
|  | Appearance (prop. normal) | Turnover (0.6/0.2 vol. hr^-1^) |  |  |  |  |  | 4.0× (*p*≤0.01) | 3.0× (*p*=0.04) |  |
|  | Bact. abund. (cells mL^-1^) | Stock. density (1.0/0.3 ml^-1^) |  | 0.3× (*p*≤0.01)^b^ |  | - | 3.3× (*p*=0.02)^b^ | - |  | - |
|  | Bact. abund. (cells mL^-1^) | Turnover (0.6/0.2 vol. hr^-1^) |  | 2.9× (*p*≤0.03) |  | - |  | - |  | - |
|  | *Vibrio* abund. (cells mL^-1^) | Turnover (0.6/0.2 vol. hr^-1^) |  |  |  | - |  | - | 0.7× (*p*=0.05) | - |
