# Supplementary material for: Coral larval aquaculture: Species-specific survival and microbial dynamics in flow-through systems
Source: PLoS One. 2026 Feb 13;21(2):e0340422. doi: 10.1371/journal.pone.0340422 (PMC12904410; doi:10.1371/journal.pone.0340422)
Supplement: S5 Table — The contrast indicates the effect size (ratio or difference) and statistical significance (p value) for each comparison. c and d refer to the comparisons of sterilization at 0.3 and 1.0 larvae mL-1 in S2 Table, respectively. (DOCX) [file pone.0340422.s012.docx]

S5 Table. Significant pairwise comparisons for main effects of treatment for larval appearance, larval settlement, temperature, and Faith’s phylogenetic diversity. The contrast indicates the effect size (ratio or difference) and statistical significance (*p* value) for each comparison. ^c^ and ^d^ refer to the comparisons of sterilization at 0.3 and 1.0 larvae mL^-1^ in Supplementary Table 2, respectively.

| Species | Response | Post hoc comparison | Contrast |
| --- | --- | --- | --- |
| *A. kenti* | Appearance (prop. normal) | All non-significant |  |
|  | Settlement (prop. settled) | Sterilization (UV / No UV) | 0.3× (*p*≤0.01)^d^ |
|  | Temperature (°C) | Sterilization (UV – No UV) | -0.2 (*p*≤0.01)^c^ -0.3 (*p*≤0.01)^d^ |
| *A. spathulata* | Settlement (prop. normal) | Turnover (0.6 / 0.2 vol. hr^-1^) | 0.3× (*p*≤0.01) |
|  | Phyl. diversity (branch length) | Turnover (0.6 – 0.2 vol. hr^-1^) | 9.2 (*p*≤0.01) |
