# Supplementary material for: Coral larval aquaculture: Species-specific survival and microbial dynamics in flow-through systems
Source: PLoS One. 2026 Feb 13;21(2):e0340422. doi: 10.1371/journal.pone.0340422 (PMC12904410; doi:10.1371/journal.pone.0340422)
Supplement: S6 Table — Responses include nitrite (NO2−), nitrate (NO3−), dissolved organic carbon (DOC), particulate carbon (Part. C), dissolved oxygen (DO), pH, total dissolved phosphorous (TDP), and particulate nitrogen (Part. N). The table includes the independent and dependent variables and the significant comparisons on each experimental day. Empty cells indicate pairwise comparisons with p > 0.05. Pairwise comparisons between treatments that differed in multiple parameters are not reported. Superscript are used to distinguish between tank conditions for multiple pairwise comparisons: a compare stocking densities without UV, b compare stocking densities with UV, c compare sterilization at 0.3 larvae mL-1, and d compare sterilization at 1.0 larvae mL-1. (DOCX) [file pone.0340422.s013.docx]

S6 Table. Significant post hoc comparisons of water quality between culture treatments for *A. kenti* (Treatment*Day and treatment main effects). Responses include nitrite ($\mathrm{NO}_{2}^{-}$), nitrate ($\mathrm{NO}_{3}^{-}$), dissolved organic carbon (DOC), particulate carbon (Part. C), dissolved oxygen (DO), pH, total dissolved phosphorous (TDP), and particulate nitrogen (Part. N). The table includes the independent and dependent variables and the significant comparisons on each experimental day. Empty cells indicate pairwise comparisons with p>0.05. Pairwise comparisons between treatments that differed in multiple parameters are not reported. Superscript are used to distinguish between tank conditions for multiple pairwise comparisons: a) compare stocking densities without UV b) compare stocking densities with UV c) compare sterilization at 0.3 larvae mL^-1^ d) compare sterilization at 1.0 larvae mL^-1^.

|  |  | Significant comparisons on each day | | | | | | |
| --- | --- | --- | --- | --- | --- | --- | --- | --- |
| Post hoc comparison | Response | -1 | 1 | 2 | 3 | 4 | 5 | 6 |
| Stocking density | $\mathrm{NO}_{2}^{-}$ (µM) |  |  | -0.1 (*p*≤0.01)^b^ | - |  | - |  |
| (1.0-0.3 mL^-1^) | $\mathrm{NO}_{3}^{-}$ (µM) | -0.3 (*p*≤0.01)^a^ -0.3 (*p*≤0.01)^b^ |  | -0.2 (*p*=0.05)^b^ | - |  | - |  |
|  | DOC (mg L^-1^) | 0.2 (*p*≤0.01)^b^ | 0.2 (*p*≤0.01)^b^ |  | - |  | - |  |
|  | Part. C (µg L^-1^) | 0.6 (*p*=0.02)^b^ |  |  | - |  | - |  |
| Turnover | DO (% sat.) | - | 2.8 (*p*≤0.01) |  |  |  |  |  |
| (0.6-0.2 vol hr^-1^) | pH | - | <0.1 (*p*=0.01) |  |  |  |  |  |
|  | $\mathrm{NO}_{2}^{-}$ (µM) |  | 0.1 (*p*≤0.01) | 0.1 (*p*≤0.01) | - |  | - |  |
|  | $\mathrm{NO}_{3}^{-}$ (µM) | 0.4 (*p*≤0.01) | 1.4 (*p*≤0.01) | 1.3 (*p*≤0.01) | - |  | - |  |
|  | DOC (mg L^-1^) | -0.3 (*p*≤0.01) |  |  | - |  | - |  |
|  | TDP (µM) |  | 0.1 (*p*≤0.01) | 0.1 (*p*≤0.01) | - |  | - |  |
|  | Part. N (µg L^-1^) |  | 21.1 (*p*≤0.01) | 9.2 (*p*=0.04) | - |  | - |  |
| Sterilization | DOC (mg L^-1^) | 0.2 (*p*≤0.01)^d^ | 0.2 (*p*≤0.01)^d^ |  | - |  | - |  |
| (UV-No UV) | $\mathrm{NO}_{2}^{-}$ (µM) | >-0.1 (*p*=0.05)^c^ |  | >-0.1 (*p*≤0.01)^d^ | - |  | - |  |
|  | $\mathrm{NO}_{3}^{-}$ (µM) |  |  | -0.2 (*p*=0.02)^d^ | - |  | - |  |
|  | TDP (µM) |  | 0.1 (*p*≤0.01)^d^ |  | - |  | - |  |
| Surface agitation | DOC (mg L^-1^) | 0.3 (*p*≤0.01) | 0.1 (*p*≤0.01) |  | - |  | - |  |
| (Ag.-No ag.) | $\mathrm{NO}_{3}^{-}$ (µM) |  | -0.4 (*p*≤0.01) |  | - |  | - |  |
|  | Part. N (mg L^-1^) |  |  | 12.1 (*p*≤0.01) | - |  | - |  |
