# Supplementary material for: Coral larval aquaculture: Species-specific survival and microbial dynamics in flow-through systems
Source: PLoS One. 2026 Feb 13;21(2):e0340422. doi: 10.1371/journal.pone.0340422 (PMC12904410; doi:10.1371/journal.pone.0340422)
Supplement: Attachment — Submitted filename: LH 2022 Reviewer comments.docx [file pone.0340422.s016.docx]

We thank the reviewers for their constructive feedback. Please find our responses below in blue following each concern. Quotations from the manuscript are *italicized*.

**Reviewer #1:**

This paper describes a multi-factorial experimental study evaluating the performance of coral larvae, water quality and microbial communities in an at-scale aquaculture setting. The study addressed multiple culture treatment conditions that would be expected to affect larval performance, including water turnover rate, UV sterilization, water surface agitation, and larval stocking density. Surprisingly, the results reported show none of these different culture conditions significantly affected larval survival, visual condition, nor settlement competence within the ranges tested, though I have some concerns with how these results are reported (or not reported) which are articulated below. The results do strongly emphasize intrinsic factors in coral larval success as the two species examined had radically different performance across all the experimental culture treatments. Meanwhile, microbial communities and some water parameters did respond to the treatments. Substantive concerns include:

1. - Ln. 110-111: Fertilization succcess is never mentioned; only that 80% of eggs were cleaving at 1.5 hr after crossing. Does this indicate that the fertilization rate was 80% or is the assumption that all were fertilized and 20% simply did not manifest cleavage yet? And was this number identical for both species in the two different months (seems unlikely)? This is a fairly important distinction (ie. whether there were 20% of unfertilized eggs incorporated (and therefore adding dead biomass) in the cultures or not) and thus fertilization needs to be reported.

We agree that the fertilization success of larval cultures can be an indicator of culture success. We used early cleavage as an indicator of fertilization and more than 80% of the eggs were fertilized in cultures of both species at 90 min (reported in lines 110-111). This is typical for these culture systems (Severati et al. 2024 J. Environ. Manag.) and *in situ* spawn slicks (Oliver and Babcock 1992 Biol. Bull.), where some eggs are exposed to sperm later than others. While we are uncertain whether the remaining eggs (<20%) were fertilized, there was no evidence of dead biomass in the cultures after 12 hrs, nor was there any evidence this affected larval survival (i.e. no steep declines in larval density) or poor water quality.

The fertilization rate is reported in the lines indicated and we have added that all eggs, fertilized or unfertilized, were added to the cultures (line 110):

*“All zygotes were then distributed amongst the 18 culture treatment tanks described below. There was no evidence that unfertilized eggs affected culture quality as no dead biomass or steep declines in larval density were observed after 12–24 hrs.”*

2. - Ln 171-175: This paragraph states events that are dismissed and not given as results, but perhaps they should be (or at the very least such dismissal needs to be clearly justified)? Four out of 18 tanks of A.kenti across three treatments essentially failed due to ‘loss of larvae through filters’. What does this mean exactly and what is the justification for dismissing that the majority of replicates of the ‘air surface agitation treatment’ failed in this way?

We tried to be clear as possible on our data handling. To further clarify, each tank was fitted with a custom 220 µm mesh filter on the outflow, allowing water exchange while retaining larvae. However, four filters leaked, resulting in the loss of larvae (as described in line 174) and invalid survival data. These four replicates were therefore excluded from larval survival analysed, as larval losses were due to equipment failure rather than mortality or treatment effects. Two of these leaks occurred in tanks assigned to the air surface agitation treatment.

We’ve clarified this statement and moved it to the Results (lines 307-309):

*“In addition, four* A. kenti *tanks were excluded from survival analysis* *due to equipment failure that caused larval losses through mesh filter leakage on the outflow which was detected during water sampling ”*

3. And more importantly, one of the treatment combinations for A.spathulata failed completely (the 2 larvae ml-l x .6 turnover). This latter point seems like a (somewhat important) result (that could provide important guidance to future efforts) but it is essentially dismissed and not even reported as a result. Statements in the results such as ‘For both species, survival was not affected by culture treatments’ do not seem strictly true given that this treatment combination failed.

We agree that reporting failed efforts is important to guide future studies. The 2 larvae ml^-1^ and 0.6 vol. hr^-1^ treatment was excluded from the results due to equipment failure within the first 24 h, which prevented data collection. This issue was described in the Methods and referenced in the Discussion (lines 528-530) to emphasise the need for further innovation in culture system design.

Following the failure, we were unable to assess survival, or any other response variables, for this treatment and all conclusions are therefore based on the remaining treatments.

We added the following clarification and have moved this statement to the beginning of the results (lines 304-306):

*“At the highest density and turnover rate tested (2 larvae mL⁻¹ and 0.6 vol. hr⁻¹), mesh filters clogged, causing overflows and losses of* A. spathulata larvae*. No data could be collected from this treatment, and it was excluded from analyses.”*

4. - Ln 187-188: Under the condition scoring, it is acknowledged in the following sentence that both ‘round’ and ‘elongated’ larval shapes are appropriate. These different shapes occur because oval larvae can be oriented either parallel or perpendicular to the plane of view in any given image. But this means a single larvae would have substantially different sizes if it was imaged in a parallel (i.e. elongate) versus in a perpendicular (i.e. round) view. Was this controlled for in the larval size measurements (i.e. were only ‘round’ or only ‘elongate’ larvae used for size measurements)?

We classified larvae as either round or elongated to exclude irregularly-shaped larvae from size measurements. Larvae were photographed in a thin layer of water in a petri dish, which prevented elongated larvae from standing upright appearing round. Typically, larvae remained round for 2-3 days post-fertilization before transitioning to an elongated form. Both morphologies were considered ‘normal’ developmental stages. As a result, reported larval sizes represent predominantly round larvae during the first 3 days and elongated larvae from days 4–7 as development progressed.

We added that the larvae were photographed “*in a thin layer of water in a petri dish to keep larvae in a flat plane*” (line 182).

5. - Fig 2: The authors mention that noise in the density measurements can account for survivorship estimates over 100%. However, I am struck with the degree of this overestimation for A.kenti (essentially all replicates and time points estimate > 100% survivorship, suggesting that the initial estimate of stocking density was perhaps substantial overestimated (as much as 50% for the kenti, surface agitation treatment which does appear to decline substantially over time (~ 150% to ~ 100%), contrary to the statistical conclusions, but I guess this is also the treatment that had only one replicate . . .) and the difference in the degree of this artifact between species. In short, the discrepancy of the A.kenti data raise concerns about the degree of artifact in (and therefore reliability) the larval density measurements.

We agree with the reviewer that survival values greater than 100% for *A. kenti* likely reflect overstocking and underestimation of initial densities relative to nominal values. Larval density was tracked as larvae mL^-1^, and even small differences (e.g., a nominal 0.3 vs measured 0.4 larvae mL^-1^) can produce large proportional changes. Measuring low densities of highly buoyant zygotes is inherently challenging; however, we are confident that the day-over-day declines observed for *A. spathulata* (and not for *A. kenti*) accurately represent the data patterns. To de-emphasise absolute survival estimates, we revised descriptions of ~100% survival for *A. kenti* to ‘high survival’ or ‘no change’ and ~40% survival for *A. spathulata* to ‘less than half’ in the Abstract and Discussion (lines 23, 24, 500).

To clarify discrepancies between nominal and measured larval densities that resulted in apparent survival estimates >100%, we added the following at the start of the Results (line 311-315):

*“The accuracy of larval stocking varied between species. For A. kenti, measured larval densities on day 2 were higher than the nominal stocking values: 0.4 (±0.02 SE) and 1.2 (±0.05 SE) larvae mL^-1^ for the nominal 0.3 and 1.0 larvae mL^-1^ treatments, respectively. In contrast, for A. spathulata, day 2 densities were lower than the nominal: 0.2 and 0.8 larvae mL^-1^ for the nominal 0.3 and 1.0 larvae mL^-1^ treatments, respectively.”*

6. Settlement assays were conducted only once and so any differences in the observed settlement (i.e., between either species or between treatments) could be due to variation in the timing of competence rather than indicating an overall lack of fitness (as is implied). This should be acknowledged in the Discussion:

This is a good point that we had not included. We acknowledged this point in the Discussion (line 528-530):

*“In this study, the different culture treatments could have influenced the time required to reach settlement competency but will require further testing to evaluate.”*

7. A few needed details and other suggestions are included below.
   - Ln 478: ‘investigated factors that influence’ probably should be re-phrased as ‘factors that were hypothesized to influence’ (since the prevailing conclusion is that they did not)

Changed as suggested.

8. Ln 492: improve should be improved.

Changed as suggested.

9. Also, this discussion confounds ‘in situ’ culture with the use of mixed-species slicks. In situ larval culture can also be conducted from single-species gamete collections (e.g., Miller et al. 2022. Rest Ecol 30: e13512 . doi: 10.1111/rec.13512).

Thank you for pointing this out. We’ve edited this paragraph to compare single-species cultures (*ex situ* or *in situ*) versus multi-species slick collections rather than in situ versus ex situ:

Line 502: Replaced ‘Coral breeding using ex situ flow-through rearing tanks’ with ‘*Coral breeding of individual species*’

Line 506: Added *‘For single-species breeding*,’ to precede ‘the benefits of controlled ex situ larval rearing…’

Line 510: added ‘[3% settlement efficiency] *for larvae from multi-species spawn slicks*’

10. Ln 501: zygote should be zygotes

Added s

11. Starting Ln 551: reference formatting switches here for a paragraph or two (and I believe the refs in this section do not appear in the list)

Thank you for identifying this error. We have fixed these references.

# **Reviewer #2:**

Review summary
Here the authors present a study investigating the effects of modifying various culture conditions on larval rearing success for two Acropora corals. Overall, the experiments and data analysis are well-designed and scientifically robust, the manuscript is highly cogent and thorough, and the results have important implications for coral larval aquaculture, making them worthy of publication. I have a few minor comments, but believe that the manuscript will be suitable for publication once these are addressed. Thank you to the authors for your important work and for making this an easy and interesting manuscript to review.

Specific comments

12. L1 — as soon as the title, and at other relevant places throughout the manuscript, the authors should clarify that the microbial communities analyzed here were from seawater samples, rather than coral-associated microbes. As of now, this is not clear until one gets to the relevant methods subsection, making some key features of the manuscript (namely, the title and abstract) somewhat confusing/unintentionally misleading.

This is a good point. We consider the title clear (‘Coral larval aquaculture: species-specific survival and microbial dynamics in flow-through systems’), but we have clarified throughout the manuscript that the microbial communities described in this study are environmental. These clarifications have been added to the Abstract, Methods, Results and Discussion sections:

Line 26: *‘microbial community composition of the culture water’*
Line 218: section heading *‘microbial community of culture water’*
Line 414: section heading *‘microbial community of culture water’*
Line 543: added *‘environmental microbial dynamics’*

13. L127–128 — using larvae from four colonies is sufficient but might mean that the results of the study do not perfectly translate to larger mixed-genotype cultures; this should be briefly mentioned in the discussion as an important caveat.

We agree that larval genetic diversity is an important consideration; however we do not believe it is a concern in this study. As noted in the preceding line, 12 colonies spawned on the first night and 13 on the second, with four colonies spawning on both nights.

For clarity, we revised this sentence to include the larval density treatments stocked on each night in parentheses. This section now reads (lines 125-130):

*“In contrast, 4.2 and 3.0 million* A. spathulata *zygotes were obtained over two consecutive spawning nights and fertilized in one tank on each night. Fifteen tanks were stocked (either 0.3 or 1.0 larvae mL^-1^) using larvae from 12 colonies on the first night, while 3 tanks were stocked (2.0 larvae mL^-1^) the following night using larvae from 13 colonies due to an insufficient number of larvae from the first night. Four colonies spawned on both nights. For A. spathulata, larvae from each fertilization tank were allocated equally across culture tanks without pooling.”*

14. L185–193 — given how many larvae were in the cultures, I am surprised at the very small sample sizes for these metrics, and feel that this should also be mentioned in the discussion as an important caveat.

We agree that larger sample sizes would strengthen the analysis; however, in some images, only a few larvae met the defined size and shape criteria for measurement. To ensure transparency, we reported the number of larvae meeting these criteria per tank each day (median=18 but range 1-64). At the treatment level, this corresponds to an average of 30-90 larval measurements per day, which we consider sufficient especially given the low variability observed in larval area.

We’ve added the total per treatment per day to the methods (line 189):

*“A median of 18 larvae (range: 1–64 larvae) was analyzed per tank per day and totalled 30-90 larvae per treatment per day.”*

15. L203–204 — please provide information regarding how this instrument was calibrated and with what frequency.

We added: “The probes were calibrated at the beginning of the 7-day measurement period according to the manufacturer’s instructions.” (line 203)

16. L256 — please specify the range and average number of reads acquired per library.

We agree that this is important information to include. We’ve added it to the microbial methods section as follows (lines 258-261):

*“Sequences from two filters per sample were pooled after preliminary inspections showed high similarity (totaling 8604-345182 sequences per sample; mean 152910). Sequence quality was assessed with FASTQC v 0.12.1 and MultiQC v 1.13a* (33, 34) *and further quality-filtered, checked for chimeras, paired reads joined, and trimmed in dada2 using default parameters (reduced to 3574-174041 sequences per sample; mean 79628).”*

17. L330–331 — fix reference.

The error seems to appear in the proofs but not the submitted document. I will double check the PDF proof for this revision.

**Reviewer #3:**

The manuscript “Coral larval aquaculture: species specific survival and microbial dynamics in flow through systems” addresses important, yet overlooked, concepts in coral aquaculture. Although there are multiple successful methods of coral husbandry are well recorded and in practice worldwide, they are seldomly reported in the scientific literature and limited to word-of-mouth knowledge and protocols within grey literature. Therefore, more experiments that tests the efficiency of different methods are required to maximize the efficiency of ex situ nurseries. This study does a good job addressing these knowledge gaps by manipulating and testing the effects of multiple environmental factors on the survival and health of coral larvae. Most of my suggestions revolve around the description of their statistical analyses in the methods and that clarifying the language in some of their statements to tighten the accuracy, consistency, and rigorousness of the study. I recommend this manuscript for publication in PLoS ONE once these comments have been addressed. Please see my specific comments outlined below:

18. Page 1, lines 49–50: “…poorly-defined…” is a strong statement that overstates the problem. Many coral nurseries have found ways to maximize their culture yields, but they are not published in peer-reviewed literature, as stated in Pollock et al. 2018. Instead, I suggest softening the language by acknowledging that maximizing larval yields has been addressed before but that there is still a need for rigorous data that determines what factors lead to maximizing those yields.

We agree and have removed ‘poorly defined’ and changed line 49 to:

“…*more work is needed to identify culture conditions that maximize larval yields*”.

19. Page 2, lines 66–68: Where did these reported survival and stocking density values come from? The literature or from preliminary analyses? Please clarify or provide the appropriate references.

“*High larval survival (70%) is achievable after three days without water exchange at 4 larvae mL⁻¹ (pH 7.3–7.6, nitrate 21–29 µmol L^−1^; 12)* ” was attributed to reference 12: *Petersen D, Hatta M, Laterveer M, Van Bergen D. Ex situ transportation of coral larvae for research, conservation, and aquaculture. Coral Reefs. 2005;24(3):510–3*. The reference to [12] was unclear in the original submission and we have removed the water quality data in the parentheses for clarity.

20. Page 6, lines 115–117: Was it 6.1 million per tank or in total for all 3 tanks? Please clarify.

We added ‘total zygotes’ for clarity.

21. Page 7, lines 137–138: Were these temperatures maintained using heaters? Please specify the equipment used to accomplish this.

We clarified how target temperatures were achieved:

*“Water temperature was controlled using a supervisory control and data acquisition system (Siemens SCADA PCS7) that mixed 22 and 36 °C seawater to reach target temperatures.”* (lines 136-138)

22. Page 8, line 161: This reference is highlighted in gray and not in numeric form. Please fix it.

Reference corrected

Page 10, line 214: Please change “was” to “were”

We didn’t change this as 250 mL of water is a single quantity that takes a singular verb, ‘was’.

23. Page 12, line 261: Please write out what ASV stands for before using the acronym.

We added *“amplicon sequence variant”* in the first instance (line 261).

24. Page 12, lines 270–272: This sentence is hard to keep track of. So each response variable was tested against multiple predictors and time within the same model, and there was a model for each response variable, correct? I would prefer you list all of the predictor variables that were incorporated in the model (the “up to 6 listed” is also vague and uninformative, since there are more than six columns) and that from now on, “culture treatment” will be used as an umbrella term for all of those variables.

We’ve clarified this sentence to emphasize that each response variable was analyzed separately and that ‘culture treatment’ refers to the combinations of larval densities, turnovers, sterilizations, and surface agitations for each species listed in Table 1.We added:

“Each response variable was analyzed separately for each species using generalized linear mixed models with culture treatment (the combinations of larval densities, turnovers, sterilizations, and surface agitations listed in Table 1), time, and the treatment*time interaction as predictors (1).” (line 273-276)

Page 12, line 274: If different error distributions were used, then they would be generalized linear models (GLMs) instead of linear models (LMs), which assume a Gaussian distribution and already have the error distribution built in . Please clarify the models that were used for the analyses.

Yes, both GLMs (other exponential distributions) and LMs (gaussian distribution) were used. We’ve changed the initial description of the models from ‘linear models’ to ‘generalized linear mixed models’. The error distribution used for each response variable is found in lines 276-279.

25. Page 12, line 277: Please specify if the binomial GLM was done using relative abundance/percentage data or binary. Binary transformations can be quite a drastic transformation, so justification should be provided along with the criteria used to determine 1s and 0s if such a transformation was actually performed. If not, then please mention that relative abundance data were used for it.

Thank you for the clarification. We’ve added that the binomial GLMs were done on proportional data.

26. Page 12, line 277–278: I assume that this was the situation for the Gaussian LM, but what diagnostic tests were used for the GLMs (i.e. normality and homogeneity of variance do not apply to GLMs with error distributions that are not Gaussian/normal)? Please clarify which diagnostics pertain to the LMs and which ones pertain to the GLMs.

Thank you for the clarification. We’ve added that the normality and homogeneity were considered for the models with gaussian error distributions while observed residuals were compared to simulated residuals for models with the other error distributions.

27. Page 13, lines 296–297: Did you use the raw relative abundance data? If not, please specify whether the data were transformed/standardized in any way to reduce the influence of extreme values.

No transformation was used on the abundance data.

Page 13, lines 303–305: If you state that there are species-level differences, please provide statistical tests that support it (i.e. a model that specifically tests variation among species). Otherwise, describe trends without implying significance. Also, please specify that these were the trends observed across all treatments.

We removed ‘there was a clear difference in survival between the two *Acropora* species” and added that these trends were consistent across all treatments. The manuscript now reads:

“*A. kenti* larvae maintained high survival (~100% of the targeted stocking density) over the 7 day culture period, whereas *A. spathulata* survival declined to less than half (~40%) by Day 7 across all treatments (Figure 2A). For both species, survival was not affected by culture treatments (Table 2).” (lines 318-321)

28. Page 14, lines 330–331: “TableError! Reference source not found” please fix this issue.

The other reviewer caught this as well. This isn’t visible in the submitted document but we will review the proofs for errors during submission of the revision.

29. Table 2 caption, line 334: You mention linear mixed models (LMEs) here, but in the main text you only refer to them as generic linear models. Please clarify which models exactly were used. If LMEs were used, please mention what you specified as your random effects in the models.

We added ‘mixed model’ to the description of the models in line 272 for consistency. A random effect of culture tank was described in line 275. Here, we also added ‘generalized linear mixed models’ to be consistent with the revisions in the Methods (see response to 23).

30. Page 16, lines 349–350: I recommend moving this statement (“Although differences in water quality were observed among culture treatments…”) to the beginning of the paragraph and merging it with the sentence that currently sits at the top of the paragraph (lines 348–349). This way, it leads more clearly into the rest of the paragraph than in the original draft.

Thank you for this suggestion. As suggested, we moved “Although differences…” to the beginning of the paragraph and moved the first sentence to the end of the paragraph.

31. Page 21, lines 436–437: Please specify which taxa in Fig. 5 belong to the classes mentioned here. I see that this is mentioned in the figure caption, but it should also be specified in the main text and in the figure legend so the reader doesn’t have to go digging for this information.

We’ve added the genera depicted in Figure 5 here as suggested.

32. Page 22, lines 474–475: Are you referring to mortality rates in the field, in the lab, or both? Please specify this and mention the hypothesized and/or observed causes of early life-stage mortality.

We agree this was ambiguous. We’ve removed the emphasis on mortality and refocussed the sentence on the larval developmental sensitivity. It now reads:

“Coral embryogenesis is a sensitive and delicate process, which can be affected by temperature (16), water quality (43), and interactions with bacteria or macroalgae (19), highlighting the need for further research into the health and survival dynamics of larvae in larger-scale rearing scenarios.”

16: Randall CJ, Szmant AM. Elevated temperature affects development, survivorship, and settlement of the elkhorn coral, Acropora palmata (Lamarck 1816). Biol Bull. 2009;217(3):269–82.

43: Richmond RH, Tisthammer KH, Spies NP. The Effects of Anthropogenic Stressors on Reproduction and Recruitment of Corals and Reef Organisms. Front Mar Sci. 2018 Jul 2;5:226.

19: Vermeij MJA, Smith JE, Smith CM, Vega Thurber R, Sandin SA. Survival and settlement success of coral planulae: independent and synergistic effects of macroalgae and microbes. Oecologia [Internet]. 2009 Mar 3;159(2):325–36. Available from: http://link.springer.com/10.1007/s00442-008-1223-7

33. Page 23, line 492: “…facilitate improve…” choose one and remove the other.

We removed ‘facilitate’.

34. Page 23, line 503: “…<1 larval mL-1…” and “…10 larval mL-1…” please change to larvae mL-1

Corrected as suggested.

35. Page 24, line 528: “with reductions of NO3–

We added ‘*in NO_3_*” here.

36. Pages 24–15, lines 551, 555, 556, 559: Update these references to numerical format.

Corrected as suggested.

37. Fig. 1: Increase font size of the legend, I also recommend labelling the different larval stocking density clusters in the diagrams or adding in miniatures of the different cluster patterns in the legend to make it easier for readers to understand and follow the figure along.

We agree and have increased the font size and used 3 colors to further distinguish the 3 stocking densities.
